# Exploring the disparity between inflammation and disability in the 10-year outcomes of people with rheumatoid arthritis

**DOI:** 10.1093/rheumatology/keac137

**Published:** 2022-03-11

**Authors:** James M Gwinnutt, Sam Norton, Kimme L Hyrich, Mark Lunt, Bernard Combe, Nathalie Rincheval, Adeline Ruyssen-Witrand, Bruno Fautrel, Daniel F McWilliams, David A Walsh, Elena Nikiphorou, Patrick D W Kiely, Adam Young, Jacqueline R Chipping, Alex MacGregor, Suzanne M M Verstappen

**Affiliations:** Centre for Epidemiology Versus Arthritis, Division of Musculoskeletal and Dermatological Sciences, Faculty of Biology, Medicine and Health, The University of Manchester, Manchester; Health Psychology Section, Institute of Psychiatry, Psychology and Neuroscience; Centre for Rheumatic Diseases, Department of Inflammation Biology, Faculty of Life Sciences and Medicine, King’s College London, London; Centre for Epidemiology Versus Arthritis, Division of Musculoskeletal and Dermatological Sciences, Faculty of Biology, Medicine and Health, The University of Manchester, Manchester; NIHR Manchester Biomedical Research Centre, Manchester University NHS Foundation Trust, Manchester Academic Health Science Centre, Manchester, UK; Centre for Epidemiology Versus Arthritis, Division of Musculoskeletal and Dermatological Sciences, Faculty of Biology, Medicine and Health, The University of Manchester, Manchester; University of Montpellier; Laboratory of Biostatistics and Epidemiology, University of Montpellier, Montpellier; Centre de Rhumatologie, Hôpital Purpan; Faculté de Médecine, Université Toulouse III, Paul Sabatier University, Inserm UMR1027, Toulouse; Department of Rheumatology, Sorbonne University—Assistance Publique Hôpitaux de Paris, Pitie Salpetriere Hospital; PEPITES team, Pierre Louis Institute of Epidemiology and Public Health, INSERM UMRS 1136, Paris, France; Pain Centre Versus Arthritis, University of Nottingham; NIHR Nottingham Biomedical Research Centre, Nottingham; Pain Centre Versus Arthritis, University of Nottingham; NIHR Nottingham Biomedical Research Centre, Nottingham; Department of Rheumatology, Sherwood Forest Hospitals NHS Foundation Trust, Sutton in Ashfield; Centre for Rheumatic Diseases, Department of Inflammation Biology, Faculty of Life Sciences and Medicine, King’s College London, London; Rheumatology Department, King’s College Hospital; Department of Rheumatology, St George’s University Hospitals NHS Foundation Trust; Institute of Medical and Biomedical Education, St George’s University of London, London; Centre for Health Services and Clinical Research, Life and Medical Sciences, University of Hertfordshire, Hatfield; Norwich Medical School, University of East Anglia; Rheumatology Department, Norfolk and Norwich University Hospitals NHS Trust, Norwich, UK; Norwich Medical School, University of East Anglia; Rheumatology Department, Norfolk and Norwich University Hospitals NHS Trust, Norwich, UK; Centre for Epidemiology Versus Arthritis, Division of Musculoskeletal and Dermatological Sciences, Faculty of Biology, Medicine and Health, The University of Manchester, Manchester; NIHR Manchester Biomedical Research Centre, Manchester University NHS Foundation Trust, Manchester Academic Health Science Centre, Manchester, UK

**Keywords:** RA, disability, epidemiology, outcomes research, psychology

## Abstract

**Objectives:**

To identify groups of people with RA with different disability trajectories over 10 years, despite comparable levels of inflammation.

**Methods:**

Data for this analysis came from three European prospective cohort studies of people with RA [Norfolk Arthritis Register (NOAR), Early RA Network (ERAN), Étude et Suivi des Polyarthrites Indifférenciées Récentes (ESPOIR)]. Participants were assessed regularly over 8 (ERAN) to 10 (NOAR/ESPOIR) years. Inclusion criteria were: recruited after 1 January 2000, <24 months baseline symptom duration, and disability (HAQ) and inflammation [two-component DAS28 (DAS28-2C)] recorded at baseline and at one other follow-up. People in each cohort also completed patient-reported outcome measures at each assessment (pain, fatigue, depressive symptoms). Group-based trajectory models were used to identify distinct groups of people with similar HAQ and DAS28-2C trajectories over follow-up.

**Results:**

This analysis included 2500 people with RA (NOAR: 1000, ESPOIR: 766, ERAN: 734). ESPOIR included more women and the participants were younger [mean (standard deviation) age: NOAR: 57.1 (14.6), ESPOIR: 47.6 (12.5), ERAN: 56.8 (13.8); women: NOAR: 63.9%, ESPOIR: 76.9%, ERAN: 69.1%). Within each cohort, two pairs of trajectories following the hypothesized pattern (comparable DAS28-2Cs but different HAQs) were identified. Higher pain, fatigue and depressive symptoms were associated with increased odds of being in the high HAQ trajectories.

**Conclusion:**

Excess disability is persistent in RA. Controlling inflammation may not be sufficient to alleviate disability in all people with RA, and effective pain, fatigue and mood management may be needed in some groups to improve long-term function.

Rheumatology key messages

- Previous research shows a disparity between long-term low inflammation and high disability in rheumatoid arthritis.
- This study identified groups with similar inflammation trajectories yet markedly different disability trajectories over 10 years.
- Pain, fatigue and depressive symptoms predicted higher disability group membership, independent of inflammation.

## Introduction

RA is a condition characterized by inflammation of the synovial joints [1]. In the past, limited treatment options were available to control this inflammation, and therefore people with RA suffered from significant pain and disability into the long term [2]. However, following the adoption of treat-to-target strategies and the widespread use of MTX for RA in the mid-1990s and subsequently the introduction of biologic treatments in the 2000s [3, 4], the ability to control inflammation drastically improved, leading to low inflammation over time for many people with RA [5, 6].

Nonetheless, this low long-term inflammation has not translated into low levels of disability. A study from the Norfolk Arthritis Register (NOAR) showed that disability followed a ‘J-shaped’ trajectory over 10 years, culminating in disability levels similar to baseline [7]. The same trajectory has been observed in other UK [6], Swedish [8] and French cohorts [9], and within a longitudinal meta-analysis [10]. Furthermore, this disparity between inflammation and disability was larger in the 2000s than in the 1990s [7, 10], despite the increasing options available to control inflammation.

Disability impacts all aspects of the lives of people with RA; higher disability is associated with reductions in work capacity [11, 12] and interference in valued life activities such as seeing friends and taking care of family [13]. Furthermore, disability is potentially a significant burden for healthcare systems. Disability is the strongest predictor of healthcare costs in RA, a finding seen across several healthcare settings [14–16]. Therefore, this excess disability despite treatment of inflammation requires investigation.

Not all individuals follow the same symptom trajectory, and the progression of many long-term outcomes important to people with RA can be described using multiple subgroups or ‘trajectory groups’ [17–19]. The hypothesis of this research project is that the disparity between inflammation and disability seen on average in cohorts of people with RA is driven by a subgroup of people with RA characterized by low-inflammation yet high disability into the long term. The aim of this analysis was to identify this subgroup within three large-scale cohort studies of people with inflammatory arthritis, two from the UK and one from France. Then, we aimed to identify factors driving the excess disability in this subgroup.

## Methods

The data for this analysis came from three inception cohorts of people with inflammatory arthritis. Participants in all three studies provided written informed consent. NOAR is a primary-care based, prospective inception cohort of people with inflammatory arthritis recruited in Norfolk, UK [20]. The inclusion criteria for NOAR are ≥2 swollen joints lasting for ≥4 weeks and being ≥16 years old. Recruitment started in 1990 and is ongoing. Participants in NOAR were assessed at baseline and then at years 1, 2, 3, 5, 7 and 10. NOAR was approved by the Cambridgeshire and Hertfordshire Research Ethics Committee (15/EE/0076).

The Early RA Network (ERAN) is a cohort of people recruited at the point of clinician diagnosis of RA from 22 outpatient rheumatology clinics in the UK and Ireland from 2002 to 2013 [21]. Participants of ERAN were seen at baseline, once between 3 and 6 months, and then annually thereafter for up to 13 years. Only the first 8 years of follow-up within ERAN were used for the current analysis due to attrition, largely driven by all centres closing to follow-up by 2018. ERAN was approved by the Trent Research Ethics Committee (01/4/047).

The *Étude et Suivi des Polyarthrites Indifférenciées Récentes* (ESPOIR) study is a cohort of people with inflammatory arthritis recruited from 14 regional centres of rheumatology across France between 2002 and 2005. The inclusion criteria for ESPOIR were >2 swollen joints lasting for >6 weeks, clinical diagnosis of RA as certain or possible, and being aged 18–70 years. Furthermore, participants were required to have received no DMARDs or glucocorticoids for >2 weeks [22]. Participants were assessed at baseline, 6 months, 12 months, 18 months, 24 months and then yearly for up to 10 years. The ESPOIR cohort study was approved by the Ethics Committee of Montpellier (020307).

For the current analysis, participants within each cohort were included if they had <24 months symptom duration at baseline, had data for disability and inflammation at baseline and one other assessment, and were recruited on or after the year 2000.

### Assessments

Participants in each study reported demographics (age, gender, smoking status, symptom duration) and completed questionnaires. Research nurses measured height and weight and performed swollen and tender joint counts at each assessment. Blood samples were taken at each assessment from which CRP was measured in NOAR and ESPOIR, and ESR in ERAN. RF (all cohorts) and anti-CCP antibody (anti-CCP; NOAR and ESPOIR only) positivity were measured from baseline blood samples. Prescription DMARD treatments were also recorded. The ERAN dataset includes the rheumatic disease comorbidity index (RDCI) [23], whereas participants of NOAR and ESPOIR self-reported comorbidities from predetermined lists (coded as 0, 1 or ≥2 comorbidities due to insufficient data to calculate RDCI). Data on baseline joint erosions were available in the ERAN and ESPOIR cohorts. X-rays were not routinely taken as part of the NOAR assessments (74.2% missing X-rays as per NOAR protocol, see Supplementary Table S1, available at *Rheumatology* online).

Global disease activity measures (such as the DAS28) include both inflammatory markers (swollen joint count, CRP/ESR) and patient-reported outcome measures [PROMs; tender joint counts, global health visual analogue scale (VAS)]. However, pharmacological treatment of RA aims to reduce inflammation, and previous research has shown that only the inflammatory components of the DAS28 were associated with MRI-detected synovitis [24]. Furthermore, this study aimed to identify specific factors driving disability in RA, such as inflammation and PROMs. As these factors are conflated in global disease activity measures, a measure of inflammation alone was needed. Therefore, in this analysis, inflammation was quantified using the two-component DAS28 (DAS28-2C). This measure combines swollen joint count and either CRP (NOAR, ESPOIR) or ESR (ERAN) using formulae designed to maximize the association between the scores and US synovitis [25]. Participants also completed several PROMs. Disability was assessed using the British [26] (NOAR, ERAN) and French [27] (ESPOIR) versions of the HAQ, with scores adjusted to account for device use. Participants of NOAR and ESPOIR completed pain and fatigue VAS, and participants of ERAN completed the Short-Form 36 (SF-36), which includes pain and fatigue (vitality) subscales [28]. Anxiety and depressive symptoms were assessed using the Arthritis Impact Measurement Scale (AIMS-2) [29] in NOAR, five variables from the French version of the AIMS-2 [30] in ESPOIR, and the mental health component of the SF36 in ERAN.

### Statistical analysis

Each cohort was analysed separately and in parallel, using the methods described below. Demographic and clinical characteristics were summarized using descriptive statistics, stratified by cohort. Within each cohort, subgroups of participants with similar HAQ and DAS28-2C trajectories were identified using multivariate group-based trajectory analysis [31], a longitudinal finite-mixture model. Specifically, this involved jointly estimating longitudinal models for both HAQ and DAS28-2C that estimated the baseline level of the outcome (intercept) and the rate of change in the outcome over time (slope), with these variables used to inform the identification of trajectory subgroups (latent classes). The number of trajectory groups was selected by assessing the Akaike and Bayesian Information Criteria, entropy and posterior probability of group membership (Supplementary Document, available at *Rheumatology* online, for details). Within pairs of trajectories that displayed the hypothesized relationship (similar inflammation but different HAQ trajectories over follow-up), baseline predictors of being in the group characterized by higher HAQ score trajectory were assessed using multivariable logistic regression. Missing data on baseline predictors were imputed using multiple imputation by iterative chained equations. Outcomes over 8 (ERAN) and 10 (NOAR and ESPOIR) years of follow-up were compared between trajectory groups using linear mixed models for continuous outcomes and generalized estimating equations (GEE) analysis for binary outcomes, controlling for age and gender. The associations between time-varying PROMs (pain, fatigue, anxiety and depressive symptoms) and disability were assessed using mixed effects models, controlling for age, gender, baseline comorbidity, baseline BMI, and HAQ score at the previous assessment. As the PROMs were measured on different scales [e.g. VAS pain (0–100) and AIMS (0–10)], to improve comparability, the PROMs were also standardized (i.e. rescaled to have mean of 0 and standard deviation of 1). Using standardized PROMs, model coefficients represent a change in the HAQ score for a standard deviation change in the PROMs (results in Supplementary File, available at *Rheumatology* online). Interaction terms were included to assess whether the association between PROMs and disability differed between trajectory groups. Trajectory analysis was performed using the traj package [32] in Stata version 14 (StataCorp: College Station, TX), and other analyses were performed using R version 3.6.0 (packages: haven [33], tidyverse [34], grid, gridExtra [35], reshape2 [36], lme4 [37], psych [38], mice [39], miceadds [40], effects [41], gee [42], broom.mixed [43]).

## Results

This analysis included 2500 people with inflammatory arthritis (NOAR = 1000, ESPOIR = 766, ERAN = 734). The ESPOIR participants were younger than the NOAR and ERAN participants [mean age (standard deviation), years: ESPOIR 47.6 (12.5); NOAR 57.1 (14.6); ERAN 56.8 (13.8)] and had a higher proportion of women (% women: ESPOIR 76.9%; NOAR 63.9%; ERAN 69.1%). The ESPOIR participants had shorter symptom duration, had more severe disease, and fewer participants were receiving csDMARDs at baseline compared with NOAR and ERAN (Supplementary Table S1, available at *Rheumatology* online).

### Group-based trajectory analysis

**Fig. 1:**
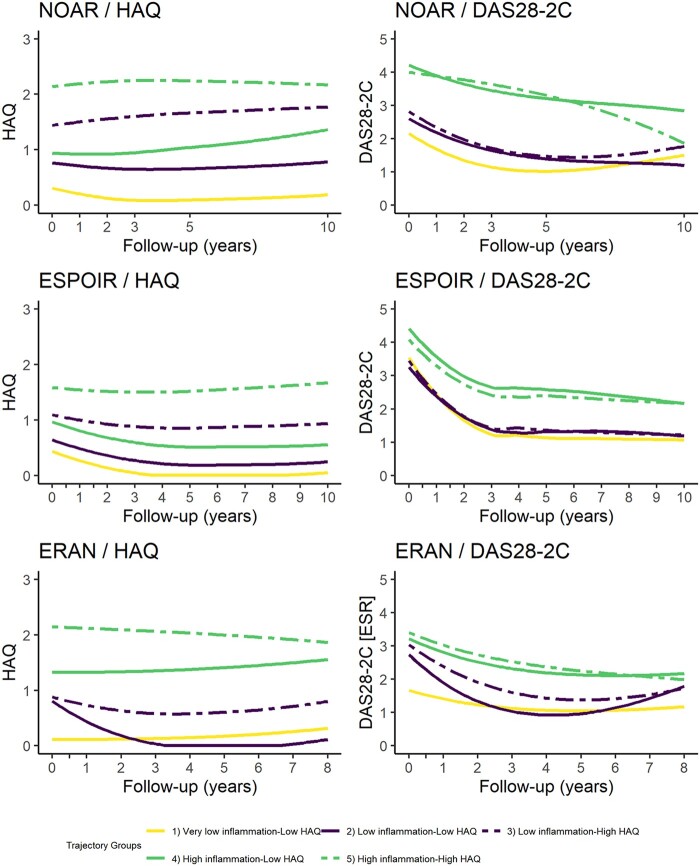
Trajectories of inflammation and disability over follow-up from the GBTM analysis The HAQ component of each trajectory group’s name is relative to the pair (i.e. in the ‘low inflammation pair’ there was one group with low HAQ and one with high HAQ). DAS28-2C: 2-component DAS; ERAN: Early RA Network, ESPOIR: Étude et Suivi des Polyarthrites Indifférenciées Récentes, GBTM: Group-Based Trajectory Model; NOAR: Norfolk Arthritis Register.

Assessment of group-based trajectory models applied to the longitudinal HAQ and DAS28-2C scores in each cohort separately resulted in the selection of a five-group trajectory model (see Supplementary Table S2 and Supplementary Figs S1–4, available at *Rheumatology* online). Each cohort contained one trajectory group with very low HAQ and DAS28-2C scores [group 1 in Fig. 1 (yellow trajectory), termed ‘Very low inflammation—Low HAQ’) (Supplementary Table S3, available at *Rheumatology* online, for baseline characteristics). In this group, the HAQ and DAS28-2C scores remained low over follow-up. The hypothesized relationship [similar inflammation (DAS28-2C) but different disability (HAQ) trajectories] was observed in two pairs of trajectories in each cohort (Fig. 1). Within each pair, the DAS28-2C scores were similar, but one trajectory had an average HAQ score of 0.5–1.0 unit higher over follow-up [groups 3 and 5 in Fig. 1 (dashed lines), termed ‘high HAQ’ trajectories] than the other trajectory [groups 2 and 4 in Fig. 1 (solid lines), termed ‘low HAQ’ trajectories] over 8–10 years. In each cohort, one pair of trajectories had lower disability and inflammation on average over the course of follow-up [groups 2 and 3 in Fig. 1 (purple trajectories), termed ‘Low inflammation pair’] compared with the other pair [groups 4 and 5 in Fig. 1 (green trajectories), termed ‘High inflammation pair’). In general, the inflammation scores of these trajectory groups improved over follow-up, whereas the HAQ scores were relatively stable. In summary, the five trajectory groups were: 1 = ‘Very low inflammation—Low HAQ’ (NOAR: 28.7%; ESPOIR: 24.3%; ERAN: 14.9%), 2 = ‘Low inflammation—Low HAQ’ (NOAR: 29.5%; ESPOIR: 29.8%; ERAN: 11.9%), 3 = ‘Low inflammation—High HAQ’ (NOAR: 19.9%; ESPOIR: 16.6%; ERAN: 28.3%), 4 = ‘High inflammation—Low HAQ’ (NOAR: 10.4%; ESPOIR: 17.8%; ERAN: 28.7%), and 5 = ‘High inflammation—High HAQ’ (NOAR: 11.5%; ESPOIR: 11.6%; ERAN: 16.2%).

### Baseline factors associated with high HAQ trajectory group membership

At baseline, participants in the ‘Low inflammation—High HAQ’ group were on average older, were more often women, had more comorbidities and had more severe pain, fatigue, anxiety and depressive symptoms compared with the ‘Low inflammation—Low HAQ’ group, despite similar inflammation (Table 1). Similar results were seen when comparing the ‘High inflammation—High HAQ’ group with the ‘High inflammation—Low HAQ’ group. Furthermore, in the low inflammation pair, the high HAQ trajectory had more erosions at baseline in ERAN and ESPOIR (data not available in NOAR). This was not seen in the high inflammation pair.

**Table 1:** Baseline characteristics of the trajectory groups

	NOAR				ESPOIR				ERAN			
	Low inflammation		High inflammation		Low inflammation		High inflammation		Low inflammation		High inflammation	
	Low HAQ	High HAQ	Low HAQ	High HAQ	Low HAQ	High HAQ	Low HAQ	High HAQ	Low HAQ	High HAQ	Low HAQ	High HAQ
*N* (%)	295 (29.5%)	199 (19.9%)	104 (10.4%)	115 (11.5%)	228 (29.8%)	127 (16.6%)	136 (17.8%)	89 (11.6%)	87 (11.9%)	208 (28.3%)	211 (28.7%)	119 (16.2%)
Age, years	55.0 (14.5)	62.8 (13.8)^**^	56.6 (12.7)	59.3 (15.2)	46.6 (13.1)	49.5 (9.9)^*^	48.7 (12.8)	52.0 (11.4)^*^	54.1 (13.5)	56.6 (14.5)	57.5 (12.6)	59.6 (13.5)
Women, *N* (%)	192 (65.1%)	138 (69.3%)	79 (76.0%)	86 (74.8%)	173 (75.9%)	109 (85.8%)^*^	99 (72.8%)	79 (88.8%)^*^	45 (51.7%)	155 (74.5%)^**^	151 (71.6%)	102 (85.7%)^*^
Symptom duration, months	7.8 (5.0)	7.8 (5.4)	8.7 (5.5)	8.7 (5.8)	3.4 (1.5)	3.7 (2.1)	3.3 (1.7)	3.7 (1.8)	10.0 (4.5)	10.0 (5.1)	10.2 (5.2)	10.1 (5.7)
BMI	26.9 (5.0)	27.7 (5.7)	28.0 (5.7)	29.3 (6.2)	24.9 (4.5)	25.7 (5.3)	25.7 (4.6)	26.5 (4.7)	26.6 (5.1)	27.3 (5.6)	28.6 (5.2)	29.2 (6.9)
BMI categories^a^												
Underweight	2 (0.7%)	6 (3.0%)	1 (1.0%)	3 (2.6%)	8 (3.5%)	3 (2.4%)	2 (1.5%)	3 (3.4%)	1 (1.1%)	4 (1.9%)	0 (0%)	3 (2.5%)
Normal weight	114 (38.6%)	60 (30.2%)	33 (31.7%)	26 (22.6%)	133 (58.3%)	63 (49.6%)	66 (48.5%)	37 (41.6%)	31 (35.6%)	69 (33.2%)	43 (20.4%)	25 (21.0%)
Overweight	107 (36.3%)	70 (35.2%)	36 (34.6%)	36 (31.3%)	56 (24.6%)	34 (26.8%)	45 (33.1%)	28 (31.5%)	30 (34.5%)	69 (33.2%)	83 (39.3%)	42 (35.3%)
Obese	70 (23.7%)	61 (30.7%)	32 (30.8%)	46 (40.0%)	31 (13.6%)	25 (19.7%)	23 (16.9%)	21 (23.6%)	15 (17.2%)	51 (24.5%)	62 (29.4%)	43 (36.1%)
Missing	2 (0.7%)	2 (1.0%)	2 (1.9%)	4 (3.5%)	0 (0%)	2 (1.6%)	0 (0%)	0 (0%)	10 (11.5%)	15 (7.2%)	23 (10.9%)	6 (5.0%)
Smoking, *N* (%)												
Smoker	68 (23.1%)	41 (20.6%)	23 (22.1%)	31 (27.0%)	110 (48.2%)	62 (48.8%)	65 (47.8%)	42 (47.2%)	28 (32.2%)	62 (29.8%)	73 (34.6%)	43 (36.1%)
Non-smoker	189 (64.1%)	137 (68.8%)	70 (67.3%)	77 (67.0%)	118 (51.8%)	65 (51.2%)	71 (52.2%)	47 (52.8%)	59 (67.8%)	143 (68.8%)	136 (64.5%)	76 (63.9%)
Missing	38 (12.9%)	21 (10.6%)	11 (10.6%)	7 (6.1%)	0 (0%)	0 (0%)	0 (0%)	0 (0%)	0 (0%)	3 (1.4%)	2 (1.0%)	0 (0%)
DAS28-CRP (ERAN: ESR)	3.6 (1.1)	3.8 (1.2)	4.5 (1.1)	4.9 (1.3)^*^	4.1 (1.1)	4.5 (1.0)^*^	5.1 (1.1)	5.1 (1.1)	4.4 (1.1)	4.6 (1.2)	4.9 (1.4)	5.5 (1.2)^**^
DAS28-2C (ERAN: ESR)	2.8 (1.4)	3.0 (1.5)	4.4 (1.3)	4.1 (1.6)	3.6 (1.2)	3.8 (1.1)	4.7 (1.3)	4.5 (1.4)	3.1 (1.2)	3.3 (1.2)	3.4 (1.4)	3.6 (1.3)
HAQ	0.8 (0.6)	1.5 (0.5)^**^	1.0 (0.5)	2.1 (0.5)^**^	0.8 (0.6)	1.2 (0.6)^**^	1.1 (0.6)	1.7 (0.6)^**^	1.0 (0.5)	0.9 (0.6)	1.3 (0.5)	2.1 (0.4)^**^
Pain VAS (ERAN: SF36-P)	36.4 (24.8)	46.8 (25.6)^**^	41.2 (23.8)	69.2 (22.2)^**^	33.5 (25.7)	45.0 (28.5)^**^	41.1 (28.0)	50.2 (26.1)^*^	47.8 (23.0)	47.0 (22.4)	40.9 (21.7)	24.3 (18.9)^**^
Fatigue VAS (ERAN: SF36-V)	42.2 (27.3)	53.0 (25.7)^**^	47.8 (28.6)	71.1 (21.9)^**^	42.9 (26.6)	53.5 (28.6)^**^	51.2 (26.0)	66.2 (23.3)^**^	46.6 (22.8)	45.0 (18.9)	36.9 (18.7)	27.4 (19.0)^**^
AIMS Depressive symptoms^b^ (ERAN: SF36-MH)	2.89 (1.91)	3.54 (1.97)^*^	3.01 (1.72)	4.85 (2.16)^**^	3.07 (1.92)	3.96 (2.01)^**^	4.03 (1.94)	5.21 (2.35)^**^	69.6 (19.6)	69.0 (18.0)	63.0 (18.0)	54.1 (20.7)^**^
AIMS Anxiety	4.00 (2.03)	4.48 (2.02)^*^	4.23 (1.68)	5.57 (2.17)^**^	4.47 (2.32)	5.32 (2.14)^**^	5.07 (2.32)	6.11 (2.30)^*^	–	–	–	–
RF, *N* (%)												
Positive	121 (41.0%)	86 (43.2%)	47 (45.2%)	39 (33.9%)	100 (43.9%)	56 (44.1%)	74 (54.4%)	40 (44.9%)	41 (47.1%)	107 (51.4%)	110 (52.1%)	77 (64.7%)^*^
Negative	164 (55.6%)	108 (54.3%)	53 (51.0%)	68 (59.1%)	128 (56.1%)	71 (55.9%)	62 (45.6%)	49 (55.1%)	34 (39.1%)	66 (31.7%)	86 (40.8%)	27 (22.7%)
Missing	10 (3.4%)	5 (2.5%)	4 (3.9%)	8 (7.0%)	0 (0%)	0 (0%)	0 (0%)	0 (0%)	12 (13.8%)	35 (16.8%)	15 (7.1%)	15 (12.6%)
Anti-CCP, *N* (%)												
Positive	90 (30.5%)	73 (36.7%)	44 (42.3%)	35 (30.4%)	84 (36.8%)	42 (33.1%)	73 (53.7%)	36 (40.4%)	–	–	–	–
Negative	187 (63.4%)	111 (55.8%)	47 (45.2%)	66 (57.4%)	144 (63.2%)	85 (66.9%)	63 (46.3%)	53 (59.6%)				
Missing	18 (6.1%)	15 (7.5%)	13 (12.5%)	14 (12.2%)	0 (0%)	0 (0%)	0 (0%)	0 (0%)				
csDMARD, *N* (%)												
Yes	155 (52.5%)	107 (53.8%)	57 (54.8%)	61 (53.0%)	13 (5.7%)	5 (3.9%)	11 (8.1%)	3 (3.4%)	57 (65.5%)	124 (59.6%)	131 (62.1%)	76 (63.9%)
No	140 (47.5%)	92 (46.2%)	47 (45.2%)	54 (47.0%)	215 (94.3%)	122 (96.1%)	125 (91.9%)	86 (96.6%)	30 (34.5%)	84 (40.4%)	80 (37.9%)	43 (36.1%)
Comorbidities (ERAN: RDCI)												
0	80 (27.1%)	34 (17.1%)	23 (22.1%)	14 (12.2%)^*^	130 (57.0%)	70 (55.1%)	79 (58.1%)	39 (43.8%)	0.41 (0.72)	0.64 (0.92)^*^	0.79 (1.1)	0.93 (1.0)
1	77 (26.1%)	46 (23.1%)	21 (20.2%)	18 (15.7%)	61 (26.8%)	36 (28.3%)	30 (22.1%)	24 (27.0%)				
≥2	57 (19.3%)	48 (24.1%)	14 (13.5%)	32 (27.8%)	37 (16.2%)	21 (16.5%)	27 (19.9%)	26 (29.2%)				
Missing	81 (27.5%)	71 (35.7%)	46 (44.2%)	51 (44.3%)	0 (0%)	0 (0%)	0 (0%)	0 (0%)				
Erosions												
Yes	–	–	–	–	20 (8.8%)	20 (15.7%)	25 (18.4%)	17 (19.1%)	19 (21.8%)	60 (28.8%)	52 (24.6%)	30 (25.2%)
No					208 (91.2%)	107 (84.3%)	111 (81.6%)	72 (80.9%)	66 (75.9%)	139 (66.8%)	149 (70.6%)	84 (70.6%)
Missing					0 (0%)	0 (0%)	0 (0%)	0 (0%)	2 (2.3%)	9 (4.3%)	10 (4.7%)	5 (4.2%)

See Supplementary Table S7, available at Rheumatology online, for proportions of missing data for each variable. ^a^BMI categories: Underweight (BMI <18.5), Normal weight (BMI ≥18.5 and <25), Overweight (BMI ≥25 and <30), Obese (BMI ≥30).

b Depression and anxiety measured with the Arthritis Impact Measurement Scales for NOAR and ESPOIR, SF36 mental health for ERAN.

* *P* < 0.05,

** *P* < 0.001 – comparing high HAQ *vs* low HAQ groups within each inflammation pair; *t*-tests used to compare continuous variables, chi^2^ for categorical variables; AIMS: Arthritis Impact Measurement Scales; Anti-CCP: anti-CCP antibodies; csDMARD: conventional synthetic DMARD; DAS28-2C: Disease Activity Score 28–2 components; ESPOIR: Étude et Suivi des Polyarthrites Indifférenciées Récentes; ERAN: Early RA Network; *N*: Number; NOAR: Norfolk Arthritis Register; SF36: Short-Form 36 (SF36-MH: SF36-mental health; SF36-P: pain scale; SF36-V: vitality scale); VAS: visual analogue scale.

Multivariable logistic regression analysis was used to identify baseline factors associated with high HAQ trajectory membership compared with low HAQ trajectory membership. Separate models were constructed for the high and low inflammation pairs (i.e. the ‘Low inflammation—High HAQ’ group was compared with the ‘Low inflammation—Low HAQ group’ and the ‘High inflammation—High HAQ’ group was compared with the ‘High inflammation—Low HAQ group’). Older age, being a woman *vs* a man, and more severe pain, fatigue and depressive symptoms were associated with increased odds of being in the higher HAQ trajectory in both the high and low inflammation pairs (Table 2; see Supplementary Table S4 and S5, available at *Rheumatology* online, for sensitivity analysis regarding missing data in NOAR). More comorbidities and serology status (NOAR: anti-CCP+; ERAN: RF+) were associated with greater odds of being in the high HAQ trajectories in NOAR and ERAN, although with wide confidence intervals that included the null for comorbidities. Erosions were associated with being in the high HAQ trajectory in the low inflammation pair in ERAN and ESPOIR, but not in the high inflammation pair, although the estimates were imprecise.

**Table 2:** Baseline predictors of high HAQ group membership compared with corresponding low HAQ group in each inflammation pair

	Low inflammation			High inflammation		
Variable	NOAR, OR (95% CI)	ESPOIR, OR (95% CI)	ERAN, OR (95% CI)	NOAR, OR (95% CI)	ESPOIR, OR (95% CI)	ERAN, OR (95% CI)
Age, years	1.07 (1.05, 1.09)	1.03 (1.01, 1.06)	1.02 (0.99, 1.04)	1.05 (1.02, 1.09)	1.03 (1.00, 1.06)	1.03 (1.01, 1.06)
Female *vs* male	1.59 (1.00, 2.52)	2.34 (1.19, 4.60)	4.15 (2.25, 7.68)	0.62 (0.25, 1.58)	3.48 (1.42, 8.57)	3.61 (1.80, 7.25)
Symptom duration, months	1.01 (0.97, 1.05)	1.09 (0.94, 1.26)	0.99 (0.93, 1.05)	0.97 (0.90, 1.04)	1.24 (1.03, 1.49)	1.01 (0.96, 1.06)
Current smoker *vs* non-smoker	1.14 (0.63, 2.06)	1.25 (0.76, 2.06)	1.01 (0.56, 1.83)	1.86 (0.65, 5.28)	1.40 (0.72, 2.72)	1.20 (0.67, 2.16)
BMI	1.03 (0.99, 1.07)	1.02 (0.97, 1.08)	1.02 (0.96, 1.09)	1.06 (0.99, 1.13)	1.05 (0.98, 1.13)	1.02 (0.98, 1.07)
Pain (VAS^a^: NOAR/ESPOIR; SF36^b^: ERAN)	1.20 (1.09, 1.31)	1.12 (1.02, 1.23)	0.88 (0.62, 1.26)	1.48 (1.23, 1.78)	1.06 (0.93, 1.20)	0.34 (0.22, 0.53)
Fatigue (VAS^a^: NOAR/ESPOIR; SF36^b^: ERAN)	1.15 (1.02, 1.28)	1.06 (0.96, 1.17)	0.90 (0.58, 1.38)	1.22 (0.96, 1.54)	1.22 (1.06, 1.41)	0.79 (0.54, 1.14)
Depressive symptoms (AIMS2: NOAR/ESPOIR; SF36^b^: ERAN)	1.11 (0.91, 1.34)	1.24 (1.07, 1.44)	1.15 (0.74, 1.80)	1.40 (1.01, 1.94)	1.14 (0.95, 1.37)	0.87 (0.62, 1.23)
Anxiety (AIMS2: NOAR/ESPOIR)	1.04 (0.86, 1.26)	1.01 (0.88, 1.15)	-	0.99 (0.67, 1.47)	1.08 (0.92, 1.27)	-
RF	0.85 (0.52, 1.41)	1.22 (0.65, 2.30)	1.53 (0.85, 2.75)	0.55 (0.21, 1.44)	0.96 (0.40, 2.31)	2.01 (1.07, 3.78)
Anti-CCP	1.79 (1.03, 3.11)	0.84 (0.43, 1.64)	-	1.21 (0.46, 3.21)	0.86 (0.35, 2.11)	-
Taking csDMARDs	0.85 (0.56, 1.31)	0.66 (0.20, 2.15)	0.77 (0.43, 1.36)	0.71 (0.30, 1.69)	0.60 (0.13, 2.77)	0.98 (0.55, 1.74)
Comorbidities (RDCI: ERAN)						
1 *vs* 0 comorbidities	1.18 (0.62, 2.26)	0.96 (0.54, 1.69)	1.42 (0.97, 2.08)	0.93 (0.31, 2.76)	1.22 (0.54, 2.75)	1.12 (0.86, 1.46)
2 *vs* 0 comorbidities	1.31 (0.73, 2.35)	0.93 (0.45, 1.91)	–	1.66 (0.53, 5.22)	1.18 (0.51, 2.74)	–
Erosions *vs* no erosions	-	1.75 (0.83, 3.68)	1.68 (0.88, 3.22)	-	1.00 (0.44, 2.29)	1.00 (0.52, 1.92)

a VAS measured in centimetres.

b SF36—higher scores indicate better status. SF36 scores were standardized, therefore odds ratio represents a standard deviation increase in SF36. AIMS2: Arthritis Impact Measurement Scales 2; Anti-CCP: Anti-CCP antibody; csDAMRD: conventional synthetic DMARD; ERAN = Early RA Network; ESPOIR = Étude et Suivi des Polyarthrites Indifférenciées Récentes; NOAR = Norfolk Arthritis Register; OR = odds ratio; RDCI = Rheumatic Disease Comorbidity Index; SF36 = Short form 36; VAS = visual analogue scale.

### Outcomes over time

The high HAQ trajectories had greater tender joint counts, pain, fatigue, depressive symptoms and anxiety than the low HAQ trajectories over follow-up in both inflammation pairs (Table 3). The high HAQ trajectories also had more comorbidities over time compared with the low HAQ trajectories across the cohorts, but with wide confidence intervals containing the null (Table 3).

**Table 3:** Comparison of the outcomes over 10 years between the high and low HAQ trajectories, stratified by inflammation pair and cohort, mean difference (95% CI)^a^

	NOAR		ESPOIR		ERAN	
	Low-inflammation: high HAQ vs low HAQ	High-inflammation: high HAQ vs low HAQ	Low-inflammation: high HAQ vs low HAQ	High-inflammation: high HAQ vs low HAQ	Low-inflammation: high HAQ vs low HAQ	High-inflammation: high HAQ vs low HAQ
Trajectory components						
DAS28-2C (NOAR/ESPOIR: CRP, ERAN: ESR)	0.17 (0.03, 0.30)	–0.13 (–0.35, 0.10)	0.04 (–0.07, 0.15)	–0.17 (–0.36, 0.02)	0.40 (0.22, 0.58)	0.11 (–0.10, 0.32)
HAQ	0.86 (0.81, 0.91)	1.22 (1.13, 1.30)	0.63 (0.59, 0.66)	0.94 (0.87, 1.01)	0.39 (0.33, 0.44)	0.72 (0.66, 0.77)
Additional PROMs						
Pain VAS/SF36–pain^b^ (ERAN)	18.0 (15.1, 21.0)	27.0 (23.1, 31.0)	11.0 (8.3, 13.8)	21.3 (17.0, 25.5)	–13.7 (–17.2, –10.2)	–15.8 (–19.0, –12.5)
Fatigue VAS/SF36–vitality^b^ (ERAN)	19.5 (15.9, 23.0)	23.4 (18.1, 28.6)	14.1 (10.6, 17.7)	22.1 (17.3, 26.9)	–10.3 (–14.4, –6.2)	–10.6 (–14.0, –7.3)
AIMS Depressive symptoms/SF36–mental health^b^ (ERAN)	1.0 (0.7, 1.3)	1.9 (1.5, 2.4)	1.2 (0.9, 1.5)	1.8 (1.3, 2.2)	–7.0 (–10.6, –3.4)	–11.8 (–15.3, –8.2)
AIMS Anxiety	0.8 (0.5, 1.1)	1.7 (1.2, 2.1)	1.2 (0.8, 1.6)	1.7 (1.2, 2.1)	–	–
DAS28 and components						
DAS28-CRP/DAS28-ESR (ERAN)	0.50 (0.37, 0.64)	0.69 (0.48, 0.91)	0.29 (0.18, 0.40)	0.44 (0.25, 0.63)	0.48 (0.28, 0.69)	0.39 (0.17, 0.61)
Swollen joint count (28)	0.35 (0.05, 0.66)	0.72 (–0.25, 1.70)	0.06 (–0.16, 0.29)	–0.57 (–1.12, –0.01)	1.13 (0.56, 1.69)	0.41 (–0.37, 1.18)
Tender joint count (28)	2.86 (2.06, 3.67)	7.22 (5.56, 8.88)	1.61 (1.03, 2.19)	3.41 (2.15, 4.67)	1.61 (0.81, 2.41)	3.14 (1.92, 4.37)
CRP/ESR (ERAN)	1.52 (–0.09, 3.12)	–4.10 (–10.11, 1.92)	0.47 (–0.68, 1.61)	2.94 (0.09, 5.78)	2.14 (–1.11, 5.40)	0.57 (–3.06, 4.19)
Comorbidities						
Presence of comorbidities^c^ (ERAN: RDCI)	OR 1.17 (0.80, 1.71)	OR 1.61 (0.93, 2.80)	OR 1.41 (0.82, 2.42)	OR 1.91 (0.87, 4.21)	0.28 (0.07, 0.49)^d^	0.07 (–0.17, 0.31)^d^

a Adjusted for age and gender.

b SF36 = higher scores indicate lower pain/fatigue/better mental health.

c NOAR and ESPOIR were analysed using generalized estimating equations analysis (GEE).

d Mean difference rather than OR for comorbidities data in ERAN, as the RDCI, which is a continuous measure, was used. DAS28-2C: 2-component DAS28; ESPOIR: Étude et Suivi des Polyarthrites Indifférenciées Récentes; ERAN: Early RA Network; NOAR: Norfolk Arthritis Register; OR: odds ratio; PROMs: Patient-Reported Outcome Measures; RDCI: Rheumatic Disease Comorbidity Index; VAS: Visual Analogue Scale.

**Fig. 2:**
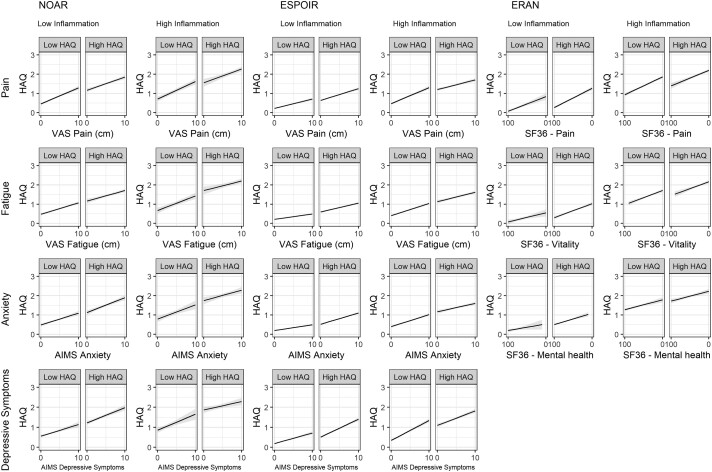
Interactions between high and low HAQ group status and patient-reported outcomes predicting HAQ score Stratified by inflammation status and cohort (unimputed results). The figure indicates that, for many PROMS, the association between PROM score and HAQ score was stronger in the lower-HAQ trajectory within each inflammation pair (i.e. the slope was steeper in the low-HAQ trajectory compared with the high-HAQ trajectory). The *x*-axis of the ERAN analyses reversed, as higher scores on the SF36 indicated better outcomes. AIMS: Arthritis Impact Measurement Scales, ERAN: Early RA network, ESPOIR: Étude et Suivi des Polyarthrites Indifférenciées Récentes, NOAR: Norfolk Arthritis Register, SF36: short form (36), VAS: visual analogue scale.

Across all trajectory groups, more severe scores on PROMs (pain, fatigue, anxiety and depressive symptoms) were all associated with increasing HAQ scores measured at the same assessment (Table 4; Supplementary Table S6, available at *Rheumatology* online, for unimputed analysis), independent of age, gender, baseline comorbidity and BMI, and HAQ at the previous assessments (see Supplementary Figure S5, available at *Rheumatology* online, for directed acyclic graph underpinning this analysis). After standardizing the PROMs to improve comparability, pain had the strongest association with HAQ (Supplementary Table S7, available at *Rheumatology* online). However, the relationship between PROMs and HAQ was different between the high and low HAQ trajectories (Fig. 2). Particularly in the high inflammation pairs, the association between the PROMs and HAQ score was stronger [i.e. the slope was steeper (interaction terms in Table 4)] in the low HAQ trajectory compared with the high HAQ trajectory (Fig. 2).

**Table 4:** Interactions between high/low HAQ trajectory group and patient-reported outcomes predicting HAQ score, stratified by inflammation pair and cohort

	Pain			Fatigue			Anxiety			Depressive symptoms		
	VAS Pain^a^ coef.	High/low HAQ coef.	Interaction	VAS Fatigue^a^ coef.	High/low HAQ coef.	Interaction	Anxiety coef.	High/low HAQ coef.	Interaction	Depressive symptoms coef.	High/low HAQ coef.	Interaction
NOAR												
Low inflammation pair	0.09 (0.08, 0.11)	0.62 (0.51, 0.72)	–0.02 (–0.04, –0.01**)**	0.05 (0.04, 0.06)	0.55 (0.44, 0.66)	0.00 (–0.02, 0.01)	0.05 (0.03, 0.07)	0.58 (0.48, 0.68)	0.00 (–0.02, 0.02)	0.06 (0.04, 0.07)	0.57 (0.48, 0.66)	0.002 (–0.02, 0.02)
High inflammation pair	0.08 (0.06, 0.10)	0.84 (0.68, 0.99)	–0.03 (–0.05, –0.002)	0.06 (0.04, 0.08)	0.89 (0.71, 1.07)	–0.02 (–0.05, 0.003)	0.05 (0.01, 0.08)	0.85 (0.69, 1.02)	–0.02 (–0.05, 0.01)	0.05 (0.01, 0.09)	0.88 (0.70, 1.07)	–0.03 (–0.06, 0.01)
ESPOIR												
Low inflammation pair	0.06 (0.05, 0.07)	0.39 (0.34, 0.44)	–0.0004 (–0.01, 0.01)	0.03 (0.03, 0.04)	0.35 (0.30, 0.41)	0.01 (0.001, 0.02**)**	0.03 (0.03, 0.04)	0.32 (0.26, 0.38)	0.02 (0.01, 0.03**)**	0.07 (0.06, 0.08)	0.31 (0.25, 0.37)	0.02 (0.01, 0.04)
High inflammation pair	0.09 (0.08, 0.10)	0.69 (0.60, 0.78)	–0.04 (–0.06, –0.03)	0.07 (0.06, 0.08)	0.66 (0.56, 0.77)	–0.02 (–0.03, –0.01)	0.07 (0.06, 0.08)	0.69 (0.58, 0.80)	–0.02 (–0.04, –0.003)	0.11 (0.09, 0.12)	0.69 (0.59, 0.79)	–0.03 (–0.05, –0.01)
ERAN	**SF36 – Pain**			**SF36 – Vitality**			**SF36 – Mental health**					
Low inflammation pair	–0.01 (–0.01, –0.01)	0.12 (–0.04, 0.27)	0.00 (–0.001, 0.003)	–0.008 (–0.01, –0.005)	0.23 (0.07, 0.38)	0.0003 (–0.002, 0.003)	–0.01 (–0.01, –0.004)	0.23 (–0.01, 0.47)	0.001 (–0.002, 0.004)	–	–	–
High inflammation pair	–0.01 (–0.01, –0.01)	0.41 (0.32, 0.51)	0.0001 (–0.002, 0.003)	–0.007 (–0.009, –0.004)	0.51 (0.40, 0.62)	–0.001 (–0.004, 0.002)	–0.004 (–0.01, –0.001)	0.57 (0.37, 0.77)	–0.002 (–0.004, 0.001)	–	–	–

Analyses controlling for age, gender, baseline comorbidity and BMI, and lagged HAQ, missing data imputed using multiple imputation.

a VAS in cm. AIMS: Arthritis Impact Measurement Scales; ERAN: Early RA network; ESPOIR: Étude et Suivi des Polyarthrites Indifférenciées Récentes; NOAR: Norfolk Arthritis Register; SF36: short form (36); VAS: visual analogue scale.

## Discussion

This large-scale analysis of 2500 people with RA with follow-up of 8–10 years illustrates the disparity between inflammation and disability for many people with this disease. This analysis identified two pairs of trajectories within each cohort, one classified as ‘high inflammation’ (groups 4 and 5) and the other classified as ‘low inflammation’ (groups 2 and 3). Each trajectory pair was characterized by a high and a low disability trajectory, despite similar inflammation scores. Therefore, 30–45% of people with RA were in groups characterized by potentially excess disability (i.e. groups 3 and 5: NOAR: 31.4%, ESPOIR: 28.2%, ERAN: 44.5%), over and above what would be expected from their level of inflammation. This excess disability is maintained up to a decade following onset. People in the higher disability trajectories were older, were more likely to be women and had worse pain, fatigue and mental health compared with the lower disability trajectories. In the high HAQ trajectories, the relationship between PROMs and disability was weaker compared with the low HAQ trajectories, particularly in the high inflammation pairs.

Our findings extend results from previous studies. A trajectory analysis of 9493 people with RA reported that 65% of those with controlled inflammation still reported persistent pain over 3 years [18]. A cohort of 232 people with early RA reported that 34% of participants had unacceptable pain at 5 years, and this was associated with lower inflammation at baseline [44]. Van der Elst *et al.* demonstrated that one in five people with rapidly and persistently controlled early RA still reported high fatigue and/or pain after 1 year of follow-up [45]. A cross-sectional analysis of 169 people with RA reported a subgroup of 57 people (33.7%) who had high pain, fatigue and depressive symptoms despite low inflammation scores [46]. In summary, for many people with RA, controlling inflammation is not sufficient to alleviate symptom burden. Our analysis illustrates these findings are consistent across several cohorts, and persist for up to a decade, with no group showing improvements in disability commencing later in follow-up, indicating once more the importance of early intervention in RA.

As PROMs (e.g. pain, fatigue, mental health) are the most consistent predictors of long-term function [47], we investigated the relationship between these PROMs and disability, both when measured at baseline and longitudinally. Baseline and time-varying pain, fatigue and mental health were all associated with high HAQ trajectory membership. In 2006, Aletaha *et al.* described reversible and irreversible components of disability in RA [48], whereby the reversible component of disability is driven by current inflammation and the irreversible component driven by joint damage and co-existing conditions. This analysis demonstrates the potential impact of comorbidities and erosion, showing that baseline erosions predicted high HAQ trajectory membership in ESPOIR and ERAN and that the high HAQ trajectories had more comorbid conditions compared with the low HAQ trajectories [49, 50]. Erosions at baseline could be as a result of treatment delays, further emphasizing the importance of early treatment in RA. Furthermore, the current analysis suggests that a third component of RA disability may comprise pain, fatigue and mental health, given the large differences in disability between pairs of trajectory groups with similar inflammation yet large differences in these PROMs. Whereas pain, fatigue and poor mental health may not be as irreversible as joint erosion, they are challenging to ameliorate in people with RA and may require both pharmacological and non-pharmacological interventions not targeting inflammation [51–53].

Despite the higher PROM scores in the high HAQ trajectories compared with the lower HAQ trajectories, a surprising result was the interaction between trajectory membership and PROMs when predicting disability. This analysis reported a weaker association between PROMs and disability in the high HAQ trajectories compared with the low HAQ trajectories. This observation could be due to the ceiling effect of the HAQ score [54], particularly as this was primarily seen in the high inflammation pair with HAQ scores nearer the top of the scale.

The strengths of this analysis include the large sample size and long-term follow-up, meaning that the disparity between inflammation and disability in people with RA over 10 years could be precisely characterized for the first time. The cohorts were well phenotyped, meaning a large array of potential factors driving disability could be assessed. However, as these were inception rather than treatment cohorts, early treatment response could not be investigated. While the inclusion criteria were similar between the three cohorts, there were several differences between the demographic and clinical characteristics of the cohorts at baseline, due to when participants were recruited (with ESPOIR’s participants recruited earlier in the disease process compared with NOAR and ERAN). The participants of ESPOIR were on average 8–10 years younger than the NOAR and ERAN cohorts. This could in part be explained by the higher rate of smoking in the ESPOIR population and the inclusion criterion of being <70 years old at baseline. While age was controlled for in the regression analyses of this project, the large difference in age between the cohorts may still be affecting comparisons between studies. There were also a number of differences in terms of the PROMs included in the three studies (i.e. the measures of pain, fatigue, mental health). A further limitation of this study was the use of different blood sample analyses across the cohorts, with ESR used in ERAN and CRP in NOAR and ESPOIR when calculating the DAS28-2C. As yet, the comparability of the DAS28-2C-ESR and DAS28-2C-CRP has not been established. Despite these limitations, the consistent results across the three cohorts suggest generalizability of the findings. While these cohorts had large samples, some effect estimates had wide confidence intervals and overlapped the null, and therefore some caution should be taken when interpreting these estimates. There was a significant proportion of missing data in some of the PROMs in the NOAR cohort, which clustered in the participants recruited earlier in the cohort and could result in bias in the reported associations. Multiple imputation was used to impute missing data, and further sensitivity analysis indicated minimal missing data bias (Supplementary Table S7, available at *Rheumatology* online). The names of the trajectory groups (i.e. high/low inflammation) are used in relation to one another, rather than based on external definitions of high and low inflammation. However, as these cohorts are representative samples of the population of people with RA, these results indicate that there is excess disability evident in RA across the spectrum of inflammation levels seen in clinical care. The scales to measure mental health across the three cohorts were necessarily brief, given the large amount of data collected at each assessment. However, they do not provide an unambiguous measurement of depressive symptoms or anxiety. Lastly, there was significant attrition over follow-up. This was particularly substantial in ERAN, meaning only 8 years of follow-up could be included.

In conclusion, this analysis illustrates that ∼30–45% of people with RA have excess disability (i.e. discordant with inflammation level), and that this excess disability is seen across inflammation levels. This excess disability is persistent, with disparity remaining at least up to 10 years following onset. People with RA in the high disability trajectories had more severe pain, fatigue and depressive symptoms compared with those in low disability trajectories, despite similar inflammation levels. This indicates the urgent need to address pain, fatigue and depressive symptoms, for example by psychological interventions for people with RA, in order to curtail long-term disability.

## Supplementary Material

keac137_Supplementary_Data

## Data Availability

The data underlying this article cannot be shared publicly for the privacy of individuals who participated in the study. The data will be shared on reasonable request to the principal investigators of each of the three datasets (NOAR: A.M., ERAN: D.A.W., ESPOIR: B.C.).
