# Supplementary material for: Exploring the disparity between inflammation and disability in the 10-year outcomes of people with rheumatoid arthritis
Source: Rheumatology (Oxford). 2022 Mar 11;61(12):4687–701. doi: 10.1093/rheumatology/keac137 (PMC9707289; doi:10.1093/rheumatology/keac137)
Supplement: keac137_Supplementary_Data [file keac137_supplementary_data.pdf]

Supplementary table 1 – Baseline characteristics of the included participants from each study

Variable	NOAR Mean (SD) § [% missing]	ESPOIR Mean (SD) § [% missing]	ERAN Mean (SD) § [% missing]	P [NOAR vs ESPOIR]	P [NOAR vs ERAN]	P [ESPOIR vs ERAN]
N	1000	766	734			
Age, years	57.1 (14.6) [0]	47.6 (12.5) [0]	56.8 (13.8) [0]	<0.0001 †	0.6831 †	<0.0001 †
Women, N (%)	639 (63.9%) [0]	589 (76.9%) [0]	507 (69.1%) [0]	<0.0001 ‡	0.0280 ‡	0.0008 ‡
Symptom duration, months	8.2 (5.4) [0]	3.4 (1.7) [0]	10.1 (5.2) [0]	<0.0001 †	<0.0001 †	<0.0001 †
BMI	27.2 (5.1) [1.6]	25.0 (4.6) [0.3]	27.8 (5.6) [8.6]	<0.0001 †	0.0178 †	<0.0001 †
BMI categories						
Underweight (BMI <18.5)	15 (1.5%)	23 (3.0%)	8 (1.1%)	<0.0001 ‡	0.2702 ‡	<0.0001 ‡
Normal weight (BMI ≥18.5 & <25)	344 (34.4%)	423 (55.2%)	207 (28.2%)			
Overweight (BMI ≥25 & <30)	372 (37.2%)	209 (27.3%)	263 (35.8%)			
Obese (BMI ≥30)	253 (25.3%)	109 (14.2%)	193 (26.3%)			
Missing	16 (1.6%)	2 (0.3%)	63 (8.6%)			
Smoking, N (%)						
Smoker	208 (20.8%)	364 (47.5%)	245 (33.4%)	<0.0001 ‡	<0.0001 ‡	<0.0001 ‡
Non-smoker	676 (67.6%)	402 (52.5%)	482 (65.7%)			
Missing	116 (11.6%)	0 (0%)	7 (1.0%)			
DAS28 (NOAR & ESPOIR: CRP, ERAN: ESR)	3.71 (1.29) [0]	4.50 (1.16) [0]	4.60 (1.43) [0]	<0.0001 †	-	-
DAS28-2C (NOAR & ESPOIR: CRP, ERAN: ESR)	3.04 (1.54) [0]	3.99 (1.31) [0]	3.13 (1.40) [0]	<0.0001 †	-	-
HAQ	0.98 (0.76) [0]	0.97 (0.69) [0]	1.12 (0.76) [0]	0.80 †	0.0001 †	<0.0001 †
Pain VAS (NOAR / ESPOIR) ; SF36-pain (ERAN)	39.3 (27.3) [0.7]	37.0 (27.5) [0.3]	45.7 (25.5) [16.6]	0.07 †	-	-
Fatigue VAS (NOAR / ESPOIR) ; SF36-vitality (ERAN)	44.7 (29.0) [33.4]	47.5 (28.1) [0.3]	42.1 (21.5) [18.7]	0.06 †	-	-
AIMS Depressive symptoms (NOAR / ESPOIR); SF36-mental health (ERAN)	3.0 (2.0) [34.3]	3.6 (2.1) [0.3]	66.3 (20.0) [18.7]	<0.0001 †	-	-
AIMS Anxiety	4.1 (2.0) [35.4]	4.9 (2.4) [0.7]	-	<0.0001†	-	-
RF, N (%))						
Positive	388 (38.8%)	354 (46.2%)	391 (53.3%)	0.013 ‡	<0.0001 ‡	<0.0001 ‡
Negative	579 (57.9%)	412 (53.8%)	247 (33.7%)			
Missing	33 (3.3%)	0 (0%)	96 (13.1%)			
Anti-CCP, N (%))						
Positive	313 (31.3%)	301 (39.3%)	-	0.029 ‡	-	-
Negative	607 (60.7%)	465 (60.7%)				
Missing	80 (8.0%)	0 (0%)				
csDMARD, N (%)						
Yes	527 (52.7%)	48 (6.3%)	471 (64.2%)	<0.0001 ‡	<0.0001 ‡	<0.0001 ‡
No	473 (47.3%)	718 (93.7%)	263 (35.8%)			
Comorbidities						
0	242 (24.2%)	446 (58.2%)	RDCI: 0.7 (1.0) [0]	<0.0001 ‡	-	-
1	245 (24.5%)	184 (24.0%)				
≥2	178 (17.8%)	136 (17.8%)				
Missing	335 (33.5%)	0 (0%)				
Erosions						
Yes	116 (11.6%)	107 (14.0%)	180 (24.5%)	-	-	<0.0001 ‡
No	142 (14.2%)	659 (86.0%)	521 (71.0%)			
Missing	742 (74.2%)	0 (0%)	33 (4.5%)			

*§ Unless otherwise stated, † t test, ‡ Chi², * Depressive symptoms and anxiety measured with the Arthritis Impact Measurement Scales for NOAR and ESPOIR, SF36 for ERAN*

Anti-CCP = anti-cyclic citrullianted protein antibodies, BMI = body mass index, csDMARD = conventional synthetic disease modifying anti-rheumatic drugs, CRP = C-reactive protein, DAS28 = Disease Activity Score 28, DAS28-2C – Disease Activity Score 28 – 2 components, ESPOIR = Étude et Suivi des Polyarthrites Indifférenciées Récentes, ESR = erythrocyte sedimentation rate, ERAN = Early Rheumatoid Arthritis Network, HAQ = Health Assessment Questionnaire, N = Number, NOAR = Norfolk Arthritis Register, RDCI = rheumatic disease comorbidity index, RF = rheumatoid factor, SD = standard deviation, SF36 = Short-Form 36, VAS = visual analogue scale

Supplementary file 2 – Comparison of different group-based trajectory models (GBTM)

Supplementary table 2 contains evaluative statistics for the multivariate GBTMs of the HAQ and DAS28-2C scores in NOAR, ESPOIR and ERAN.

Supplementary table 2 – Evaluative statistics of the GBTMs in NOAR, ESPOIR and ERAN

Cohort	Trajectory groups, N	AIC	BIC	Entropy	Average posterior probability of group membership
**NOAR**	2	11738.9	11806.0	0.92	0.96
	3	11245.5	11344.4	0.85	0.94
	4	11071.2	11201.9	0.77	0.91
	5	10967.2	11129.7	0.71	0.88
	6	10883.2	11077.5	0.67	0.87
	7	10853.0	11079.0	0.60	0.84
	8	10787.8	11045.6	0.60	0.84
**ESPOIR**	2	19410.0	19482.7	0.93	0.97
	3	18797.2	18904.4	0.87	0.95
	4	18528.1	18669.7	0.80	0.92
	5	18294.6	18470.6	0.76	0.90
	6	18157.6	18368.1	0.71	0.89
	7	18166.6	18411.5	-*	-*
	8	17948.2	18227.6	0.69	0.88
**ERAN**	2	9966.0	10031.7	0.90	0.96
	3	9685.0	9781.9	0.79	0.92
	4	9549.3	9677.3	0.70	0.88
	5	9503.8	9663.0	0.64	0.85
	6	9381.3	9571.6	0.60	0.84
	7	9364.7	9586.1	0.55	0.82
	8	9257.8	9510.4	0.54	0.83

***** Model failed with 7 trajectory groups
AIC = Akaike Information Criterion, BIC = Bayesian Information Criterion, ERAN = Early Rheumatoid Arthritis Network, ESPOIR = Étude et Suivi des Polyarthrites Indifférenciées Récentes, N = number, NOAR = Norfolk Arthritis Register

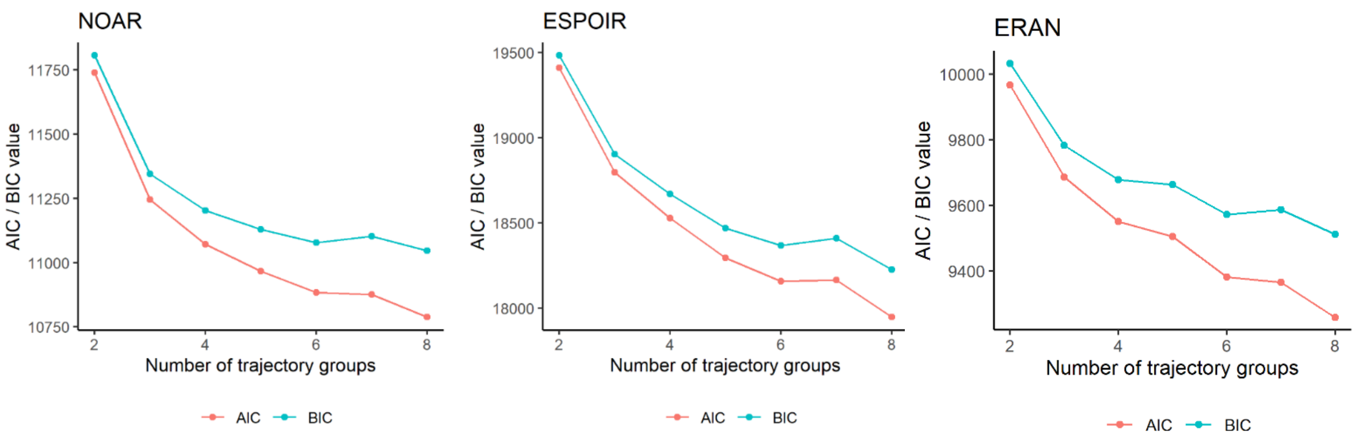

Supplementary Figure 1 – The AIC and BICs from GBTMs with different numbers of trajectories for NOAR, ESPOIR and ERAN

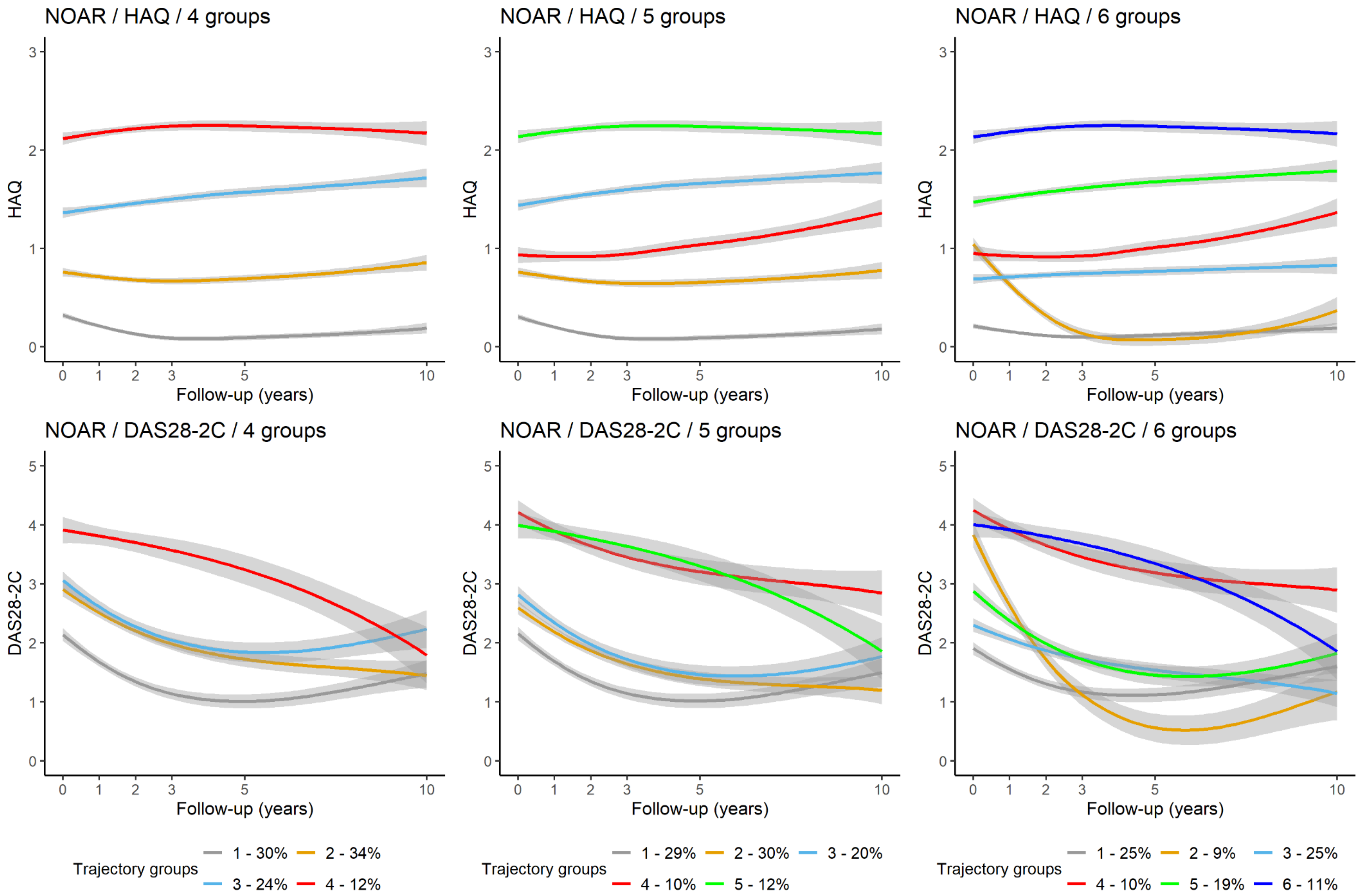

Supplementary Figure 2 – 4 /5 /6 trajectory group models in NOAR

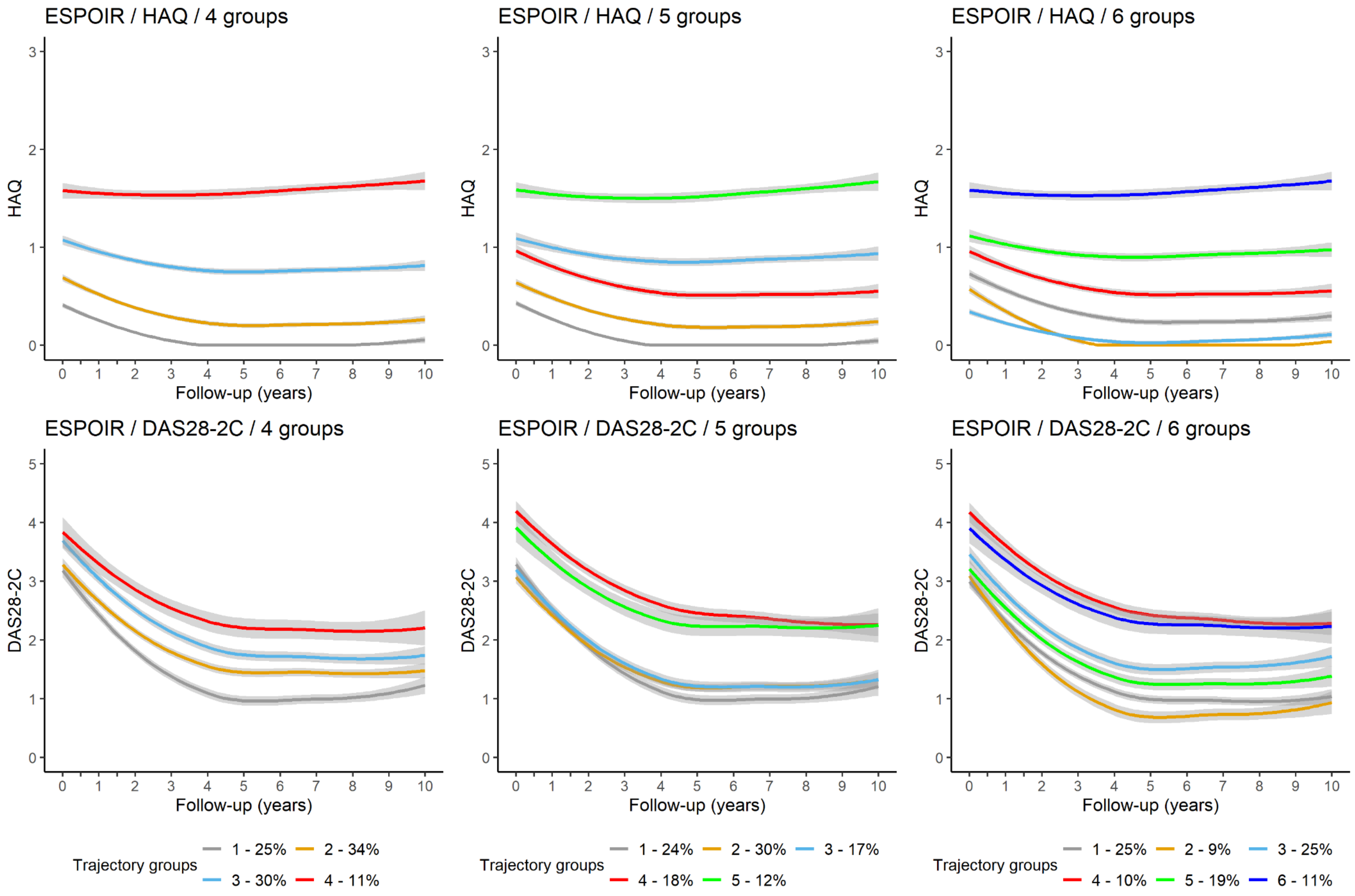

Supplementary Figure 3 – 4 /5 / 6 trajectory group models in ESPOIR

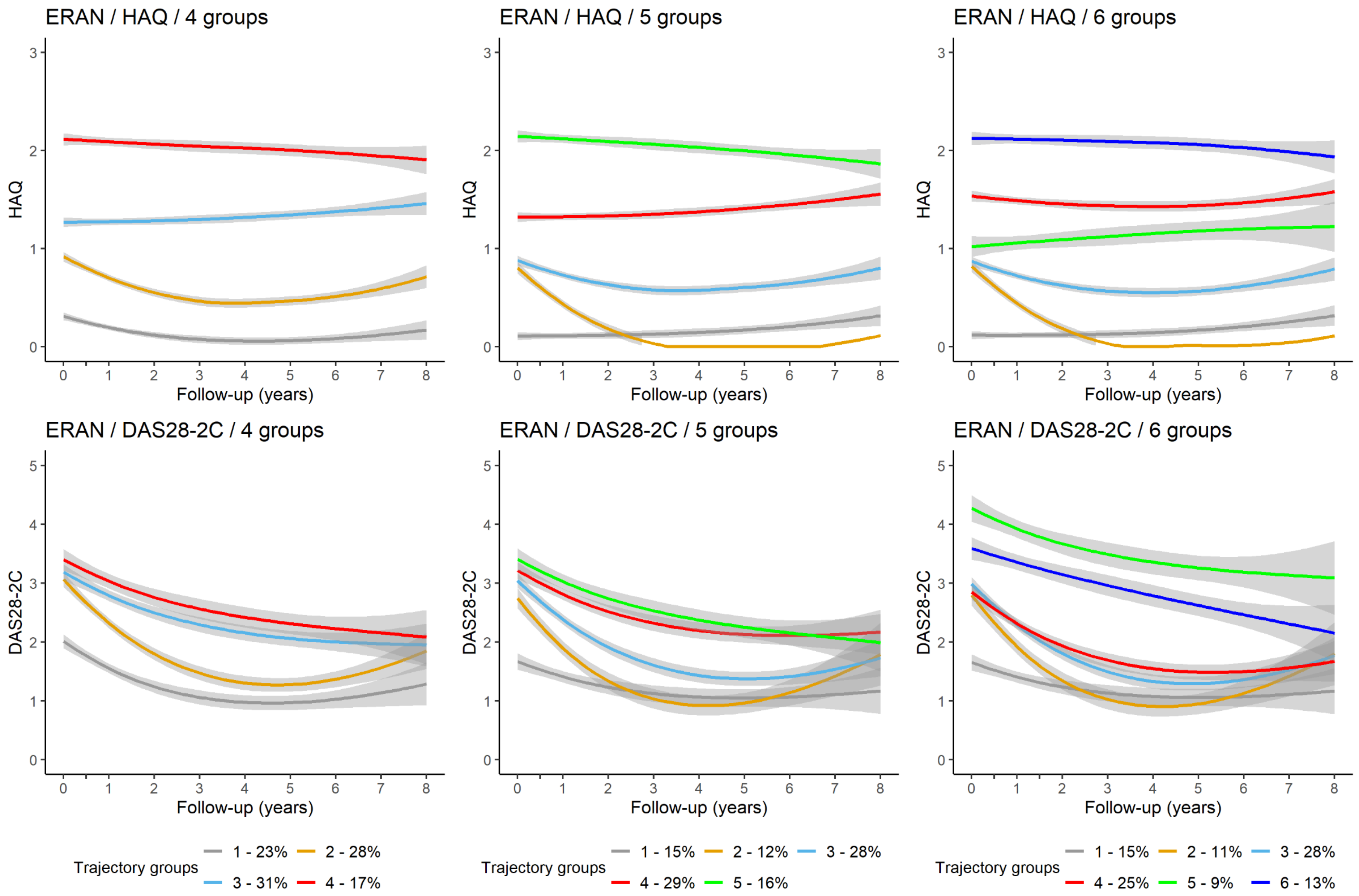

Supplementary Figure 4 – 4 /5 / 6 trajectory group models in ERAN

Supplementary table 2 and Supplementary figure 1 illustrate that for NOAR and ESPOIR, the AIC and BIC curves begin to plateau at 6 trajectory groups. For ERAN, the first plateau is at 4 groups, however, there are continued substantial decreases in AIC and BIC to 6 groups.

The entropy and the average posterior probability of group membership continue to deteriorate for each additional trajectory group included, for both NOAR and ESPOIR and particularly for ERAN. Therefore, a trade-off between model fit and trajectory group classification appears to favour a model with 4-6 trajectories.

Examining the five group model for NOAR (Supplementary figure 2), there are two pairs of trajectories that follow the hypothesised pattern (similar inflammation scores [DAS28-2c] but different HAQ). Trajectories 4 (red) and 5 (green) have similar DAS28-2C scores but different HAQ. The same is true for trajectories 2 (tan) and 3 (turquoise), although these groups have lower inflammation and disability scores overall. Comparing the NOAR 5 group model to the NOAR 6 group model, the only difference is an additional small group (group 2) which starts with high DAS28-2c and then has a large decrease early on to low levels. This same rapid decline is seen in the HAQ scores of this group. A similar pattern is seen when comparing the group allocations between models with different numbers of trajectory groups from the ESPOIR dataset (Supplementary Figure 3) and the ERAN dataset (Supplementary Figure 4).

In conclusion, most participants are allocated to similar groups in models with different numbers of trajectories. Each time a new group is added, a new small trajectory group emerges, shared from two groups in the model with one fewer group. Therefore, this indicates a preference for a more parsimonious model. Therefore, to achieve an optimal balance between model fit and group allocation as well as favouring a more parsimonious model, a 5 group trajectory model was selected.

Supplementary Table 3 – Baseline characteristics of the Very low inflammation-Low HAQ group, stratified by cohort

	NOAR, Mean (SD) / N(%) [% missing]	ESPOIR Mean (SD) / N(%) [% missing]	ERAN Mean (SD) / N(%) [% missing]
N	287	186	109
Age, years	54.5 (14.6) [0]	44.6 (12.9) [0]	55.0 (14.7) [0]
Women, N (%)	144 (50.2%) [0]	129 (69.4%) [0]	54 (49.5%) [0]
Symptom duration, months	8.6 (5.6) [0]	3.2 (1.7) [0]	10.7 (5.0) [0]
BMI	26.0 (3.9) [2.1]	23.6 (3.8) [0]	26.8 (4.4) [8.3]
BMI categories §			
Underweight	3 (1.1%)	7 (3.8%)	0 (0%)
Normal weight	111 (38.7%)	124 (66.7%)	39 (35.8%)
Overweight	123 (42.9%)	46 (24.7%)	39 (35.8%)
Obese	44 (15.3%)	9 (4.8%)	22 (20.2%)
Missing	6 (2.1%)	0 (0%)	9 (8.3%)
Smoking, N (%)			
Smoker	45 (15.7%)	85 (45.7%)	39 (35.8%)
Non-smoker	203 (70.7%)	101 (54.3%)	68 (62.4%)
Missing	39 (13.6%)	0 (0%)	2 (1.8%)
DAS28-CRP (ERAN:ESR)	3.0 (1.0) [0]	4.3 (1.2) [1.1]	3.1 (1.2) [0]
DAS28-2C (ERAN: ESR)	2.4 (1.4) [0]	3.9 (1.3) [0]	1.8 (1.2) [0]
HAQ	0.4 (0.5) [0]	0.6 (0.6) [0]	0.1 (0.2) [0]
Pain VAS (ERAN: SF36)	24.8 (22.3) [0.7]	26.2 (24.4) [0.5]	72.4 (20.2) [11.0]
Fatigue VAS (ERAN: SF36)	32.6 (27.8) [30.0]	37.4 (27.4) [0.5]	59.3 (18.2) [16.5]
AIMS Depressive symptoms (ERAN: SF36)	2.23 (1.58)	3.09 (1.94)	78.8 (17.5) [16.5]
AIMS Anxiety	3.47 (1.84) [31.7]	4.35 (2.31) [0]	-
RF, N (%))			
Positive	95 (33.1%)	84 (45.2%)	56 (51.4%)
Negative	186 (64.8%)	102 (54.8%)	34 (31.2%)
Missing	6 (2.1%)	0 (0%)	19 (17.4%)
Anti-CCP, N (%))			
Positive	71 (24.7%)	66 (35.5%)	-
Negative	196 (68.3%)	120 (64.5%)	
Missing	20 (7.0%)	0 (0%)	
csDMARD, N (%)			
Yes	147 (51.2%)	16 (8.6%)	83 (76.1%)
No	140 (48.8%)	170 (91.4%)	26 (23.9%)
Comorbidities [ERAN: RDCI)			
0	91 (31.7%)	128 (68.8%)	0.51 (0.80)
1	83 (28.9%)	33 (17.7%)	
≥2	27 (9.4%)	25 (13.4%)	
Missing	86 (30.0%)	0 (0%)	
Erosions			
Yes	-	25 (13.4%)	19 (17.4%)
No		161 (86.6%)	83 (76.1%)
Missing		0 (0%)	7 (6.4%)

*§ BMI categories: Underweight (BMI <18.5), Normal weight (BMI ≥18.5 & <25), Overweight (BMI ≥25 & <30), Obese (BMI ≥30); * Depressive symptoms and anxiety measured with the Arthritis Impact Measurement Scales for NOAR and ESPOIR, SF36 for ERAN*

AIMS = Arthritis Impact Measurement Scales, Anti-CCP = anti-cyclic citrullianted protein antibodies, BMI = body mass index, csDMARD = conventional synthetic disease modifying anti-rheumatic drugs, DAS28 = Disease Activity Score 28, DAS28-2C – Disease Activity Score 28 – 2 components, ESPOIR = Étude et Suivi des Polyarthrites Indifférenciées Récentes, ESR = erythrocyte sedimentation rate, ERAN = Early Rheumatoid Arthritis Network, HAQ = Health Assessment Questionnaire, N = Number, NOAR = Norfolk Arthritis Register, RF = rheumatoid factor, SD = standard deviation, SF36 = Short-Form 36, VAS = visual analogue scale

Supplementary Table 4 – Proportion of missing data for each of the baseline characteristics reported in Table 1 of the manuscript

	NOAR				ESPOIR				ERAN			
	Low inflammation		High inflammation		Low inflammation		High inflammation		Low inflammation		High inflammation	
	Low HAQ, % missing	High HAQ, % missing	Low HAQ, % missing	High HAQ, % missing	Low HAQ, % missing	High HAQ, % missing	Low HAQ, % missing	High HAQ, % missing	Low HAQ, % missing	High HAQ, % missing	Low HAQ, % missing	High HAQ, % missing
Age, years	0	0	0	0	0	0	0	0	0	0	0	0
Women	0	0	0	0	0	0	0	0	0	0	0	0
Symptom duration, months	0	0	0	0	0	0	0	0	0	0	0	0
BMI	0.7	1.0	1.9	3.5	0	1.6	0	0	11.5	7.2	10.9	5.0
Smoking	12.9	10.6	10.6	6.1	0	0	0	0	0	1.4	0.9	0
DAS28-CRP (ERAN:ESR)	0	0	0	0	2.2	2.4	0.7	1.1	0	0	0	0
DAS28-2C (ERAN: ESR)	0	0	0	0	0	0	0	0	0	0	0	0
HAQ	0	0	0	0	0	0	0	0	0	0	0	0
Pain VAS (ERAN: SF36-P)	0	1.5	0	1.7	0.4	0	0	0	10.3	21.2	18.5	15.1
Fatigue VAS (ERAN: SF36-V)	27.5	35.7	44.2	43.5	0.4	0	0	0	13.8	23.1	19.4	15.1
AIMS Depressive symptoms* (ERAN: SF36-MH)	28.1	37.2	45.2	43.5	0	0.8	0	1.1	13.8	23.1	19.4	15.1
AIMS Anxiety	28.5	39.2	46.2	46.1	0.4	1.6	0	2.3	-	-	-	-
RF	0.3	0.3	0.4	0.7	0	0	0	0	13.8	16.8	7.1	12.6
Anti-CCP	6.1	7.5	12.5	12.2	0	0	0	0	-	-	-	-
csDMARD	0	0	0	0	0	0	0	0	0	0	0	0
Comorbidities	27.5	35.7	44.2	44.3	0	0	0	0	0	0	0	0
Erosions	-	-	-	-	0	0	0	0	2.3	4.3	4.7	4.2

** Depressive symptoms and anxiety measured with the Arthritis Impact Measurement Scales for NOAR and ESPOIR, SF36 mental health for ERAN*

AIMS = Arthritis Impact Measurement Scales, Anti-CCP = anti-cyclic citrullianted protein ntibodies, BMI = body mass index, csDMARD = conventional synthetic disease modifying anti-rheumatic drugs, DAS28 = Disease Activity Score 28, DAS28-2C – Disease Activity Score 28 – 2 components, ESPOIR = Étude et Suivi des Polyarthrites Indifférenciées Récentes, ESR = erythrocyte sedimentation rate, ERAN = Early Rheumatoid Arthritis Network, HAQ = Health Assessment Questionnaire, N = Number, NOAR = Norfolk Arthritis Register, RF = rheumatoid factor, SD = standard deviation, SF36 = Short-Form 36 (SF36-MH = SF36-mental healthSF36-P = pain scale, SF36-V = vitality scale), VAS = visual analogue scale

There was a high proportion of missing data on some of the PROMs in NOAR (see Supplementary Table 4) and these missing data clustered in the participants recruited earlier to the cohort. Therefore, in sensitivity analysis, the predictors analysis was performed in the NOAR cohort in those people recruited on or after 2004. As can be seen when comparing Supplementary Table 5 with Table 2 in the manuscript, there were minimal differences in the odd ratios of the PROMs, indicating minimal missing data bias.

Supplementary Table 5 – Baseline predictors of being in the high HAQ group from multivariable logistic regression, only including NOAR patients recruited after 1.1.2004

**Variable**	**NOAR low severity, OR (95% CI)**	**NOAR high severity, OR (95% CI)**
Age, years	1.07 (1.05, 1.10)	1.04 (1.00, 1.09)
Female vs male	1.45 (0.83, 2.52)	0.42 (0.12, 1.44)
Symptom duration, months	1.04 (0.99, 1.09)	0.97 (0.88, 1.08)
Current smoker vs non-smoker	1.34 (0.64, 2.79)	4.22 (0.92, 19.32)
BMI	1.04 (1.00, 1.10)	1.14 (1.02, 1.26)
Pain VAS†	1.20 (1.08, 1.35)	1.53 (1.20, 1.96)
Fatigue VAS†	1.13 (1.01, 1.27)	1.18 (0.92, 1.51)
Depressive symptoms (AIMS2)	1.06 (0.88, 1.28)	1.46 (1.03, 2.06)
Anxiety (AIMS2)	1.08 (0.90, 1.29)	0.91 (0.62, 1.35)
RF	0.83 (0.44, 1.56)	1.01 (0.26, 3.99)
Anti-CCP	1.99 (1.00, 3.98)	0.65 (0.15, 2.80)
Taking csDMARDs	0.88 (0.52, 1.47)	0.61 (0.19, 1.93)
Comorbidities (RDCI: ERAN) 1 vs 0 comorbidities	1.31 (0.69, 2.48)	0.69 (0.19, 2.49)
2 vs 0 comorbidities	1.45 (0.74, 2.84)	2.50 (0.57, 11.06)

† VAS measured in centimetres

AIMS2 = Arthritis Impact Measurement Scales 2, Anti-CCP = Anti-cyclic citrullinated peptide antibody, BMI = body mass index, CI = confidence interval, csDAMRD = conventional synthetic disease modifying anti-rheumatic drug, HAQ = Health Assessment Questionnaire, NOAR = Norfolk Arthritis Register, OR = odds ratio, RDCI = Rheumatic Disease Comorbidity Index, RF = rheumatoid factor, SF36 = Short form 36, VAS = visual analogue scale

Supplementary Table 6 – Unimputed results from interactions between high / low HAQ trajectory group and patient reported outcomes predicting HAQ score, stratified by inflammation pair and cohort

	Pain			Fatigue			Anxiety			Depressive Symptoms		
	**VAS Pain§ coef.**	**High/low HAQ coef.**	**Interaction**	**VAS Fatigue§ coef.**	**High/low HAQ coef.**	**Interaction**	**Anxiety coef.**	**High/low HAQ coef.**	**Interaction**	**Depressive Symptoms coef.**	**High/low HAQ coef.**	**Interaction**
**NOAR**												
Low inflammation pair	0.08<br>(0.07, 0.10)	0.71<br>(0.60, 0.82)	**-0.02**<br>**(-0.03, 0.00)**	0.06<br>(0.05, 0.07)	0.69<br>(0.56, 0.82)	-0.01<br>(-0.02, 0.01)	0.06<br>(0.04, 0.08)	0.65<br>(0.51, 0.78)	0.02<br>(-0.01, 0.04)	0.06<br>(0.04, 0.07)	0.66<br>(0.55, 0.78)	0.02<br>(-0.01, 0.04)
High inflammation pair	0.09<br>(0.07, 0.11)	0.85<br>(0.58, 1.08)	-0.02<br>(-0.05, 0.01)	0.08<br>(0.05, 0.10)	1.04<br>(0.77, 1.28)	-0.03<br>(-0.06, 0.01)	0.07<br>(0.04, 0.10)	0.96<br>(0.70, 1.19)	-0.02<br>(-0.06, 0.02)	0.08<br>(0.05, 0.11)	1.01<br>(0.78, 1.21)	-0.04<br>(-0.08, 0.004)
**ESPOIR**												
Low inflammation pair	0.05<br>(0.04, 0.06)	0.41<br>(0.37, 0.45)	**0.01**<br>**(0.00, 0.02)**	0.03<br>(0.02, 0.03)	0.38<br>(0.33, 0.43)	**0.02**<br>**(0.01, 0.03)**	0.03<br>(0.02, 0.04)	0.32<br>(0.26, 0.38)	**0.03**<br>**(0.02, 0.04)**	0.05<br>(0.04, 0.06)	0.33<br>(0.27, 0.38)	**0.04**<br>**(0.02, 0.05)**
High inflammation pair	0.08<br>(0.07, 0.09)	0.73<br>(0.64, 0.81)	**-0.03**<br>**(-0.05, -0.02)**	0.06<br>(0.05, 0.07)	0.72<br>(0.62, 0.82)	**-0.01**<br>**(-0.03, 0.00)**	0.06<br>(0.05, 0.07)	0.78<br>(0.66, 0.89)	**-0.02**<br>**(-0.04, -0.00)**	0.10<br>(0.09, 0.11)	0.75<br>(0.64, 0.85)	**-0.03**<br>**(-0.05, -0.01)**
**ERAN**	**SF36 - Pain**			**SF36 - Vitality**			**SF36 – Mental Health**					
Low inflammation pair	-0.01<br>(-0.01, -0.01)	0.43<br>(0.24, 0.62)	**-0.002**<br>**(-0.005, 0)**	-0.005<br>(-0.007, -0.002)	0.48<br>(0.27, 0.68)	**-0.003**<br>**(-0.006, 0)**	-0.003<br>(-0.006, 0)	0.56<br>(0.25, 0.87)	**-0.002**<br>**(-0.006, 0.001)**	-	-	-
High inflammation pair	-0.01<br>(-0.01, -0.01)	0.34<br>(0.22, 0.45)	0.001<br>(-0.001, 0.004)	-0.007<br>(-0.009, -0.005)	0.45<br>(0.33, 0.57)	0.00<br>(-0.003, 0.003)	-0.005<br>(-0.007, -0.003)	0.45<br>(0.26, 0.63)	0<br>(-0.002, 0.003)	-	-	-

Analyses controlling for age, gender, baseline comorbidity and BMI, and lagged HAQ

§ VAS in cm

AIMS = Arthritis Impact Measurement Scales, ERAN = Early rheumatoid arthritis network, ESPOIR = Étude et Suivi des Polyarthrites Indifférenciées Récentes, HAQ = Health Assessment Questionnaire, NOAR = Norfolk Arthritis Register, SF36 = short form (36), VAS = visual analogue scale

Supplementary Table 7 – Standardised results from interactions between high / low HAQ trajectory group and PROMS predicting HAQ score, stratified by inflammation pair and cohort

	**Pain**			**Fatigue**			**Anxiety**			**Depressive symptoms**		
	**VAS Pain§**	**High/low HAQ coef.**	**Interaction**	**VAS Fatigue§**	**High/low HAQ coef.**	**Interaction**	**Anxiety§**	**High/low HAQ coef.**	**Interaction**	**Depressive symptoms§**	**High/low HAQ coef.**	**Interaction**
**NOAR**												
Low inflammation pair	0.23 (0.20, 0.27)	0.53 (0.45, 0.62)	**-0.05 (-0.09, -0.02)**	0.14 (0.11, 0.18)	0.55 (0.47, 0.62)	0.00 (-0.04, 0.04)	0.10 (0.07, 0.13)	0.58 (0.52, 0.64)	0.00 (-0.04, 0.04)	0.11 (0.08, 0.14)	0.58 (0.51, 0.65)	0.00 (-0.04, 0.05)
High inflammation pair	0.20 (0.15, 0.26)	0.71 (0.58, 0.83)	**-0.07 (-0.13, -0.00)**	0.17 (0.11, 0.23)	0.77 (0.60, 0.94)	-0.06 (-0.13, 0.01)	0.11 (0.03, 0.18)	0.77 (0.62, 0.91)	-0.04 (-0.11, 0.02)	0.12 (0.03, 0.21)	0.78 (0.65, 0.92)	-0.06 (-0.14, 0.01)
**ESPOIR**												
Low inflammation pair	0.13 (0.11, 0.15)	0.39 (0.35, 0.43)	-0.001 (-0.03, 0.02)	0.09 (0.07, 0.11)	0.39 (0.35, 0.43)	**0.03 (0.00, 0.05)**	0.08 (0.06, 0.10)	0.39 (0.35, 0.43)	**0.04 (0.01, 0.07)**	0.14 (0.12, 0.15)	0.37 (0.33, 0.41)	**0.04 (0.02, 0.07)**
High inflammation pair	0.24 (0.22, 0.27)	0.56 (0.49, 0.63)	**-0.11 (-0.15, -0.08)**	0.19 (0.17, 0.22)	0.57 (0.50, 0.64)	**-0.06 (-0.10, -0.02)**	0.17 (0.14, 0.19)	0.59 (0.53, 0.66)	**-0.05 (-0.10, -0.01)**	0.24 (0.22, 0.27)	0.57 (0.51, 0.64)	**-0.07 (-0.11, -0.03)**
**ERAN**	**SF36 - Pain**			**SF36 - Vitality**			**SF36 – Mental Health**					
Low inflammation pair	-0.27 (-0.31, -0.22)	0.17 (0.10, 0.23)	0.02 (-0.03, 0.07)	-0.16 (-0.22, -0.10)	0.24 (0.17, 0.31)	0.01 (-0.05, 0.06)	-0.12 (-0.18, -0.06)	0.28 (0.22, 0.33)	0.01 (-0.04, 0.07)	-	-	-
High inflammation pair	-0.18 (-0.23, -0.13)	0.43 (0.36, 0.51)	0.01 (-0.05, 0.07)	-0.13 (-0.18, -0.09)	0.49 (0.41, 0.58)	-0.01 (-0.07, 0.05)	-0.08 (-0.13, -0.02)	0.48 (0.41, 0.55)	-0.03 (-0.09, 0.03)	-	-	-

Analyses controlling for age, gender, baseline comorbidity and BMI, and lagged HAQ

§ Scales standardised to allow easier comparison – coefficient represents change in HAQ score for each standard deviation increase in the PROM

AIMS = Arthritis Impact Measurement Scales, ERAN = Early rheumatoid arthritis network, ESPOIR = Étude et Suivi des Polyarthrites Indifférenciées Récentes, HAQ = Health Assessment Questionnaire, NOAR = Norfolk Arthritis Register, PROM = patient reported outcome measures, SF36 = short form (36), VAS = visual analogue scale

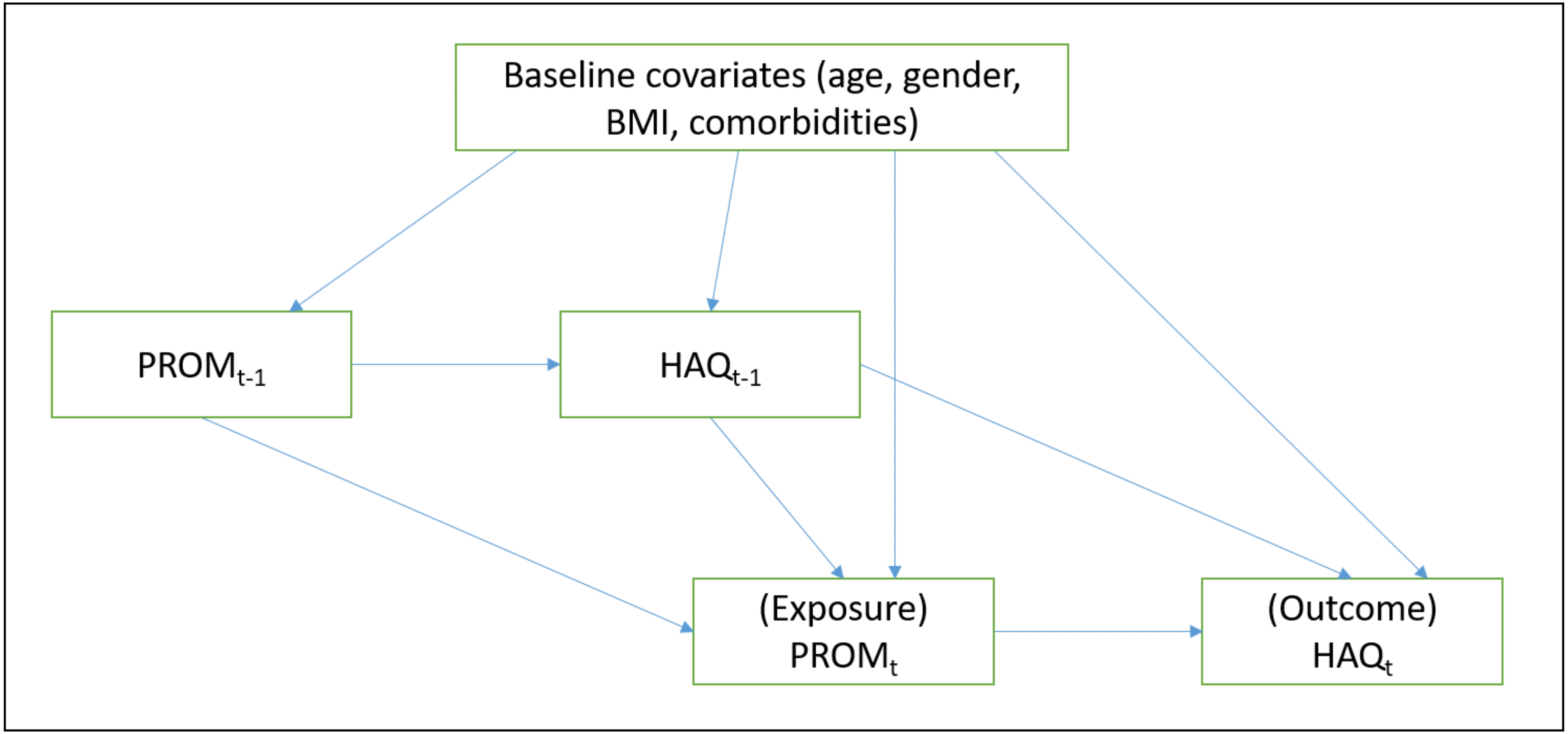

Supplementary Figure 5 – Directed Acyclic Graph illustrating the minimal adjustment set (previous HAQ score and baseline covariates) for the relationship between current patient reported outcome measure (PROM) and disability (HAQ)

Daggity code to reproduce the above DAG on http://www.dagitty.net/

```
dag {

bb="0,0,1,1"

"Baseline covariates" [pos="0.568,0.189"]

"HAQ (t-1)" [pos="0.425,0.386"]

"PROM (t-1)" [pos="0.189,0.386"]
```

```
HAQ [outcome,pos="0.744,0.606"]

PROM [exposure,pos="0.461,0.601"]

"Baseline covariates" -> "HAQ (t-1)"

"Baseline covariates" -> "PROM (t-1)"

"Baseline covariates" -> HAQ

"Baseline covariates" -> PROM

"HAQ (t-1)" -> HAQ

"HAQ (t-1)" -> PROM

"PROM (t-1)" -> "HAQ (t-1)"

"PROM (t-1)" -> PROM

PROM -> HAQ

}
```
